# The impact of para-aortic lymph node irradiation on disease-free survival in patients with cervical cancer: A systematic review and meta-analysis

**DOI:** 10.1016/j.ctro.2022.05.006

**Published:** 2022-05-30

**Authors:** Leslie J.H. Bukkems, Ina M. Jürgenliemk-Schulz, Femke van der Leij, Max Peters, Cornelis G. Gerestein, Ronald P. Zweemer, Peter S.N. van Rossum

**Affiliations:** a Department of Radiation Oncology, University Medical Center Utrecht, Utrecht University, Utrecht, The Netherlands; b Department of Gynecologic Oncology, University Medical Center Utrecht, Utrecht University, Utrecht, The Netherlands

**Keywords:** Cervical cancer, Chemoradiotherapy, Para-aortic radiotherapy, Disease-free survival, Meta-analysis

## Abstract

**Background:**

Patients with locally advanced cervical cancer without para-aortic lymph node metastases (PAO-LNM) at diagnosis who undergo concurrent chemoradiotherapy are at 4–11% risk of developing PAO-LNM during follow-up. Some studies suggest a beneficial influence of elective para-aortic radiotherapy (PAO-RT) on disease-free survival (DFS) in these patients. The aim of this study was to systematically review and meta-analyse literature on the impact of PAO-RT on DFS in cervical cancer patients.

**Methods:**

A systematic search of PubMed/MEDLINE and EMBASE databases was performed. The analysis included intervention studies that reported on DFS in patients with cervical cancer who received chemotherapy and pelvic radiotherapy with or without PAO-RT. From each included study, relevant study characteristics and outcome data including the hazard ratio (HR) adjusted for potential confounders were extracted. An overall pooled adjusted hazard ratio (aHR) for DFS after PAO-RT versus no PAO-RT was calculated using a random-effects model.

**Results:**

A total of 2,016 articles were evaluated. Eleven articles were included in the systematic review, of which 3 were appropriate for quantitative meta-analysis. Pooling of these 3 cohorts (including 1,113 patients) demonstrated a statistically significant association between PAO-RT and DFS (pooled aHR 0.87, 95% confidence interval: 0.79–0.97). No significant heterogeneity among reported aHRs was observed (I^2^ = 0.0%).

**Conclusions:**

This meta-analysis suggests a modest but significant beneficial impact of elective para-aortic radiotherapy on DFS in patients with locally advanced cervical cancer who undergo concurrent chemoradiotherapy. This finding based on non-randomized studies provides an imperative for further investigation in prospective controlled trials.

## Highlights

• Standard of care of locally advanced cervical cancer is based on concurrent chemoradiotherapy.
• Para-aortic radiotherapy (PAO-RT) has become controversial in the chemotherapy era.
• Systematic review on impact of PAO-RT on disease-free survival yielded 11 studies.
• Meta-analysis of 3 studies showed pooled adjusted HR of 0.87 (95% CI: 0.79–0.97).
• Findings support further investigation in prospective controlled trials.

## Introduction

Although screening has led to a major decline in the number of cases, uterine cervical cancer remains the fourth most common cancer in women [1]. Lymph nodes play an important role in the metastatic progression of cervical cancer and the presence of lymph node metastasis is the single most important prognostic factor [2]. Lymphatic spread often follows a predictable order, starting at the regional pelvic lymph nodes, followed by the para-aortic lymph nodes and, finally, distant metastases [3]. The para-aortic lymph nodes are also a common site for disease recurrence after pelvic radiotherapy with concurrent chemotherapy for locally advanced cervical cancer [4], [5], [6].

At diagnosis, approximately 25–47% of patients with cervical cancer have pelvic lymph node metastasis, whereas 4–8% of patients present with para-aortic lymph node metastases (PAO-LNM) [7], [8], [9]. Patients with pelvic lymph node metastasis are at increased risk of developing para-aortic lymph node metastases (PAO-LNM) [5], [7]. In fact, the multicenter EMBRACE (intErnational MRI-guided BRAchytherapy in CErvical cancer) study group showed that in patients with pelvic nodal metastases at diagnosis the risk of developing PAO-LNM during follow-up is 11%, as opposed to 4% in patients with cN0 disease at diagnosis [8], [10]. Also, the RetroEMBRACE and EMBRACE-I studies indicated that para-aortic failure is the major challenge for nodal control [11].

Para-aortic lymph nodes are generally assessed with magnetic resonance imaging (MRI) and/or ^18^F-fluordeoxyglucose positron emission tomography with integrated computed tomography (^18^F-FDG PET-CT). However, with these imaging modalities occult PAO-LNM may be missed in a considerable amount (12%) of patients [12]. In patients with suspicious pelvic lymph nodes, the false-negative rate of imaging for PAO-LNM is as high as 22% (versus 9% in patients without suspicious pelvic lymph nodes) [13]. Therefore, in patients without evidence of PAO-LNM, extending the standard pelvic radiation field to cover the para-aortic lymph node area is sometimes considered to treat potentially present occult PAO-LNM. A recent analysis from the multicenter EMBRACE study group showed that the addition of elective para-aortic radiotherapy (PAO-RT) was associated with significantly less para-aortic nodal failure, especially in patients with pelvic lymph node metastases [10]. No information on disease-free survival (DFS; including recurrences other than PAO-LNM) was provided in that study. The disadvantage of adding PAO-RT is the increased risk of complications, such as enteritis, diarrhea, and myelosuppression, compared to pelvic radiotherapy only [14], [15].

Before concurrent chemotherapy became a standard addition to radiotherapy, patients with locally advanced cervical cancer received definitive radiotherapy alone. In 1995, the Radiation Therapy Oncology Group (RTOG) reported that prophylactic PAO-RT was associated with improved survival rates [16]. However, in the current era of concurrent chemoradiotherapy in which chemotherapy also targets occult metastatic disease (as supported by the observed association between the number of chemotherapy cycles and metastasis-free survival [17], [18], the role of elective PAO-RT has become more controversial. Therefore, the aim of this study was to systematically review and meta-analyse the available literature on the impact of adding elective PAO-RT to standard pelvic radiotherapy on disease-free survival (DFS) in patients with locally advanced cervical cancer and pelvic lymph node involvement who are treated with concurrent chemoradiotherapy.

## Methods

This review has been registered in the PROSPERO international database of prospectively registered systematic reviews, accessible at https://www.crd.york.ac.uk/prospero/ with registration number CRD42021275325. This study was reported according to the PRISMA 2020 guideline for reporting systematic reviews [19].

### Search strategy

A systematic literature search was conducted and last updated on 5 November 2021 to identify all studies from the year 1995 onwards reporting on PAO-RT and DFS or overall survival (OS) in patients with locally advanced cervical cancer. The year 1995 was chosen as this was the publication year of the long-term results of the key RTOG-7920 trial on PAO-RT [16] and studies before that date were considered to have low generalizability due to outdated staging and treatment techniques. The search terms ‘cervix’, ‘cancer’, ‘para-aortic’, ‘radiotherapy’, and synonyms, were used to search Embase and PubMed/MEDLINE databases according to the search strategy presented in Table 1.

**Table 1:** Search strategy and results.

No.	**Search query**	**PubMed**	**Embase**
#1	Cancer* OR carcinoma* OR tumor* OR tumour* OR malignan* OR neoplasm*	4,635,340	6,348,518
#2	Radiotherapy OR radiation OR irradiation OR X-ray therapy	724,640	1,109,237
#3	Cervical OR cervix	280,564	377,381
#4	Para-aortic OR paraaortic OR *peri*-aortic OR periaortic OR PALN OR retroperitoneal	38,290	64,827
#5	**#1 AND #2 AND #3 AND #4**	**1,022**	**2,138**

### Study selection

After removal of duplicates, all titles and abstracts were evaluated for eligibility. Any citation considered potentially relevant was retrieved for full text review. Full texts of potentially relevant articles were then evaluated for inclusion.

Eligibility criteria included studies that reported on radiotherapy of para-aortic lymph nodes or extended field radiotherapy in cervical cancer patients with pelvic lymph node metastases, that provided data on DFS and/or OS. At least 1 patient should have had pelvic lymph node metastases in both the group with PAO-RT and the group without PAO-RT. Subsequently, studies in which patients received no concurrent chemotherapy were excluded, as well as reviews, editorials, letters to the editor, conference abstracts and case reports, and studies with less than 5 cervical cancer patients. In case multiple articles showed significant overlap, only the article describing the largest study population was included. Publications written in languages other than English or Dutch were excluded. Finally, reference lists of included articles were screened for other potentially relevant articles.

### Data extraction and quality assessment

From each included study, relevant treatment and outcome data and study characteristics were extracted. Information on the author, year of publication, country, study design, number of participants, International Federation of Gynaecology and Obstetrics (FIGO) tumour stage, age of the patients, lymph node metastasis, treatment regimen, follow-up duration and survival data were captured. DFS in the studies was defined as the time after primary treatment that the patient survived without recurrence of disease in any location (i.e. local, regional or distant). In accordance with recent large-scale data from EMBRACE, reported 5-year DFS rates in the studies were expected to be in the order of 65–70% [20].

The primary outcome measure was the hazard ratio (HR) for DFS after PAO-RT versus no PAO-RT that in case of non-randomized studies had to be adjusted for potential confounders in either propensity score matching, inverse probability treatment weighting (IPTW) or multivariable analysis. This adjusted HR (aHR) was chosen in accordance with the Cochrane Handbook for Systematic Reviews recommendations [21], because this represents the least biased within-study estimate of the DFS impact of PAO-RT (in contrast to unadjusted HR or crude survival point estimates). Overall survival (OS) was not chosen as primary outcome as –compared to DFS– it was considered a more indirect outcome of extending versus not extending the radiation field to PAO lymph nodes, with DFS being the more direct potentially affected outcome. Study quality and possible bias of articles included in the meta-analysis were assessed using the Cochrane Risk Of Bias In Non-randomized Studies of Interventions tool (ROBINS-I) [22] and visualized using the associated risk-of-bias visualization tool (robvis) [23].

### Statistical analysis

Reported independent associations between PAO-RT and DFS, expressed as aHRs with 95% confidence intervals (95% CIs), were pooled using a random-effects model. Heterogeneity across aHRs of individual studies was assessed using the I^2^ statistic. An I^2^ between 30 and 60% was considered moderate heterogeneity according to the Cochrane Handbook for Systematic Reviews [24]. The number of studies was insufficient for properly powered subgroup analyses or meta-regression. A sensitivity analysis was performed through exclusion of 1 study from the meta-analysis to determine the effect on the overall pooled aHR.

In the synthesis of all the studies (including those with no reported aHR), 3-year and 5-year DFS and OS estimates were summarized as median values as well as mean values weighted for study sample size. Importantly, these summary estimates represent crude (univariable) survival estimates that were not adjusted for confounders, and should therefore be regarded of inferior validity compared to the pooled aHR estimate. All analyses were performed using R 4.0.3 software (The R Foundation for Statistical Computing, Vienna, Austria; ‘metafor’ package).

## Results

### Identification of studies

**Fig. 1:**
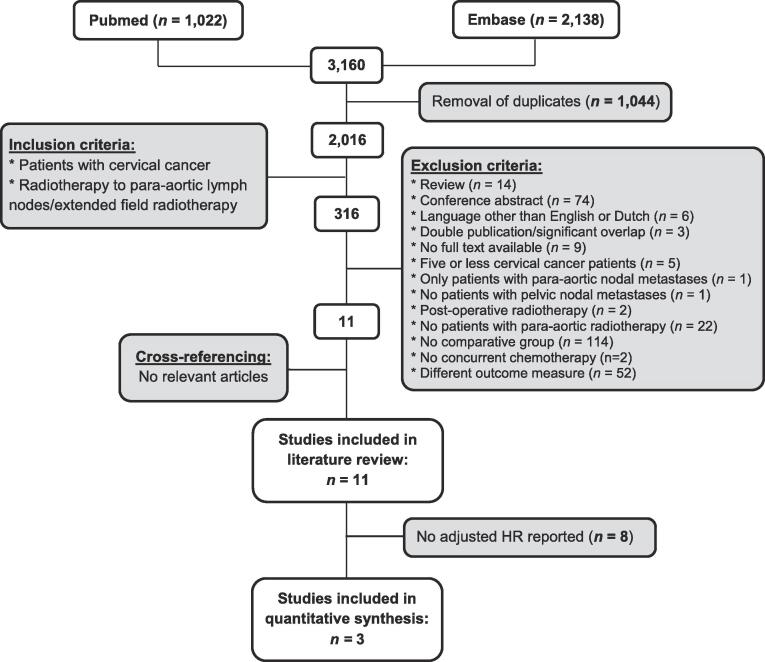
Flowchart summarizing search results and study selection.

The selection of studies is demonstrated in Fig. 1. The search strategy yielded 3,160 articles. After removal of duplicates (n = 1,044), 2,016 articles remained of which 316 were selected for full text screening. After full text screening, 305 articles were excluded for various reasons (Fig. 1), including lack of a comparative group without PAO-RT (n = 110) or insufficient data in conference abstracts (n = 73). Other major reasons for exclusion were the lack of data on DFS or OS (n = 49) or on a patient group receiving PAO-RT (n = 20). Two studies were excluded because no concurrent chemotherapy was administered [16], [25]. One study was excluded because it only included cervical cancer patients without pelvic lymph node metastasis [26]. Cross-referencing did not yield any additional articles. Eleven articles remained eligible for inclusion in the systematic review, of which 3 were appropriate for quantitative meta-analysis with pooling of aHRs. The 8 studies excluded from quantitative analysis reported no HR for DFS.

### Study characteristics

Characteristics of the 11 studies included in the literature analysis are summarized in Table 2. Four [27], [28], [29], [30] out of 11 studies were prospective by nature and 7 were retrospective [31], [32], [33], [34], [35], [36], [37]. No eligible studies published before the year 2014 were found, and 7 out of 11 included studies were published in or after 2016. Most studies reported using MRI [33], [34], both CT and MRI [30], [31], [32] or a combination of ^18^F-FDG PET, CT and MRI [27], [28], [29], [36], [37] for nodal staging. In all studies, the majority of patients was treated with concurrent platinum-based chemotherapy. Total radiation dose administered to the para-aortic region ranged from 40 Gy to 50.4 Gy.

**Table 2:** Characteristics of studies included for literature analysis.

**Study, year**	**Design**	**FIGO stage**	***n* total**	***n*** **PAO-RT**	***n*** **no PAO-RT**	***n* (%) PLN + in PAO-RT**	***n* (%) PLN + in no PAO-RT**	**Mean PAO-RT dose**
**Al Asiri 2014**[27]	Prosp	IIB-IVA	74	38	36	23 (60.5)	15 (41.7)	45 Gy
**Liang 2014**[28]	Prosp	IB2-IIIB	79	32	47	32 (100.0)	5 (10.6)	40 Gy
**Park 2014**[31]	Retro	IB2-IIIB	203	88	115	59 (67.0)	34 (29.6)	45 Gy
**Yap 2014**[32]	Retro	NR	228	73	155	46 (63.0)	21 (13.5)	40 Gy
**Kim 2016**[29]	Prosp	IB1-IVA	114	57	57	NR	NR	45 Gy
**Ouyang 2017**[33]	Retro	I-III	107	55	52	44 (80.0)	25 (48.1)	45 Gy
**Lee 2017**[34]	Retro	IB2-IVA	206	96	110	45 (46.9)	32 (29.1)	50.4 Gy
**Oh 2017**[35]	Retro	IB1-IVA	126	52	74	52 (100.0)	74 (100.0)	45 Gy
**Wang 2018**[36]	Retro	IB-IVA	778	154	624	88 (57.1)	95 (15.2)	50.4 Gy
**Sanders 2021**[37]	Retro	IB1-IVA	96	49	47	49 (100.0)	47 (100.0)	45–50.4 Gy
**Huang 2021**[30]	Prosp	IB1-IVA	34	17	17	17 (100.0)	17 (100.0)	40–45 Gy

NR: Not reported. PAO-RT: para-aortic radiotherapy. PLN+: pelvic lymph node metastases. Prosp: prospective. Retro: retrospective.

Detailed data on pelvic lymph node involvement was available for 10 studies [27], [28], [30], [31], [32], [33], [34], [35], [36], [37] and 3 of those studies only included patients with pelvic lymph node metastasis [30], [35], [37]. The average proportion of patients with positive pelvic lymph node status in PAO-RT groups versus no-PAO-RT groups across the 11 studies was 69.6% vs. 28.6%, respectively.

Additionally, data on para-aortic lymph node involvement was provided by all 11 studies, and -in accordance with the domain of the current review- 10 studies excluded patients with suspicious lymph nodes. One study did include a few patients with suspicious para-aortic lymph nodes [33]. In that study, the proportion of patients with suspicious para-aortic lymph nodes in the PAO-RT group was 11 of 55 (20%) versus none in the no-PAO-RT group [33]. Pathologic (PAO) nodes received a simultaneous integrated boost to 60 Gy in that study [33]. The study was nevertheless included in this review as in its multivariable analysis for DFS the influence of PAO-RT was adjusted for the most distant level of involved lymph nodes.

Crude DFS data were provided in all studies. Three studies [32], [33], [36] including a total of 1,113 patients provided an adjusted HR for DFS and were eligible for quality assessment and quantitative meta-analysis.

### Quality assessment

The results of the quality assessment are presented in Fig. 3. Overall, the studies included in the meta-analysis were of moderate quality. All studies included a consecutive series of patients with appropriate exclusion criteria, but moderate concern in patient selection was present in 1 study that included few patients with para-aortic metastases at baseline (but adjusted for this confounder in multivariable analysis) [33]. Selection bias was of particular concern in the studies because patients with positive lymph node status or a generally higher risk of occult para-aortic lymph node metastases were more likely to receive PAO-RT. However, all 3 studies included in the meta-analysis used multivariable Cox regression analysis in an attempt to adjust for these confounders [32], [33], [36]. One of these studies in addition attempted to adjust for selection bias due to baseline differences by conducting propensity score matching [36].

There were no reported deviations from intended interventions, and no concerns regarding missing data, classification of interventions or measurement of outcomes. Bias in selection of the reported result was of moderate concern in all 3 studies, as no pre-registered protocol or statistical analysis plan was provided. As such, to some extent the retrospective nature of the studies may have resulted in selective multivariable modelling and reporting of results.

### Quantitative synthesis

**Fig. 2:**
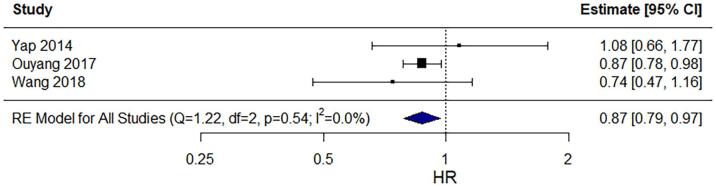
Forest plot of the pooled analysis of 3 studies reporting adjusted HRs of the association of para-aortic radiotherapy versus no para-aortic radiotherapy with disease-free survival.

Three studies provided adjusted HRs for DFS [32], [33], [36]. All 3 studies were retrospective by nature. In total, 1,113 patients were included, of which 282 (25.3%) received PAO-RT. All 3 studies used a Cox proportional hazards model to adjust for the effect of clinical covariates tumor size and pelvic lymph node metastasis [32], for tumor size, highest level of involved lymph nodes, treatment duration, and nadir-haemoglobin levels [33], or for tumor size, pelvic lymph node metastasis, histology, and FIGO stage [36]. As a separate analysis, 1 study additionally used propensity score matching to adjust for potential confounders, including age, histology, FIGO stage, tumor size, and lymph node involvement [36]. Pooled analysis across the 3 studies that provided an aHR demonstrated a statistically significant beneficial DFS with an overall pooled aHR of 0.87 (95% CI: 0.79–0.97; Fig. 2). The I^2^ statistic revealed no significant heterogeneity in aHR estimates among the 3 studies (I^2^ = 0.0%).

**Fig. 3:**
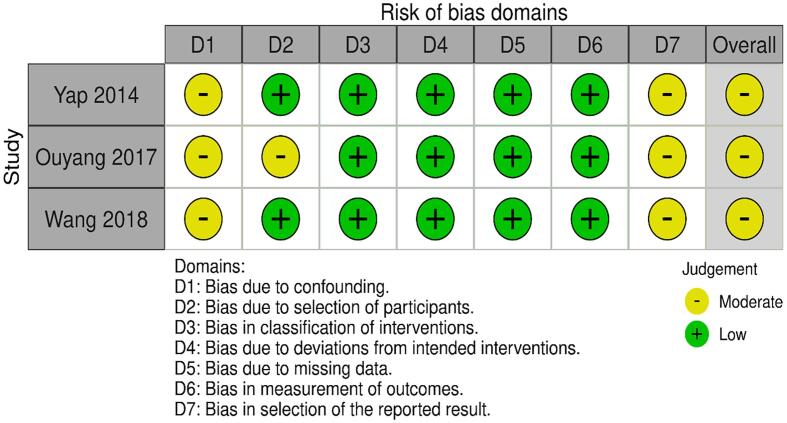
A graphical overview of the risk of bias assessment in 3 studies included in the meta-analysis.

Sensitivity analysis was performed by excluding the 1 study that inappropriately included 11 patients with PAO nodal metastases at baseline [33], although these 11 patients only represented less than 1% of the total 1,113 patients in the primary meta-analysis. Pooling of the 2 remaining studies resulted in a similar overall pooled aHR for DFS of 0.88, but the statistical significance was lost (i.e. 95% CI: 0.61–1.27).

Crude (univariable) survival outcome data as reported in all 11 studies included in this systematic review are summarized in Table 3. At 3 years, median reported DFS among 10 studies was 80.6% versus 71.0% for patients who received PAO-RT versus no PAO-RT, respectively. Weighted for study sample size, the average 3-year DFS across studies was 74.0% versus 70.3%, respectively. At 5 years, median reported DFS among 9 studies was 76.0% versus 68.1%, respectively. Weighted for study sample size, the average 5-year DFS across studies was 72.0% versus 67.3%, respectively. No further quantitative synthesis or significance testing of these studies was performed due to the lack of adjustment for confounders in these estimates.

**Table 3:** Crude (univariable) survival outcome data of studies on patients who received para-aortic radiotherapy versus no para-aortic radiotherapy.

	**Disease-free survival (DFS)**				**Overall survival (OS)**			
	**3-year**		**5-year**		**3-year**		**5-year**	
**Study, year**	**PAO-RT**	**No PAO-RT**	**PAO-RT**	**No PAO-RT**	**PAO-RT**	**No PAO-RT**	**PAO-RT**	**No PAO-RT**
**Al Asiri 2014**[27]	83.0%	78.0%	80.3%	69.1%	75.0%	73.0%	72.4%	60.4%
**Liang 2014**[28]	82.0%	54.0%	79.0%	46.5%	87.0%	62.0%	86.0%	48.0%
**Park 2014**[31]	NR	NR	75.8%	74.5%	81.0%	84.0%	71.7%	74.8%
**Yap 2014**[32]	49.0%	73.0%	47.0%	67.0%	66.0%	84.0%	NR	NR
**Kim 2016**[29]	88.0%	74.0%	85.4%	71.2%	93.0%	85.0%	82.2%	81.9%
**Ouyang 2017**[33]	61.0%	73.7%	NR	NR	79.4%	82.3%	NR	NR
**Lee 2017**[34]	80.5%	71.0%	79.6%	69.6%	87.8%	79.0%	87.8%	74.5%
**Oh 2017**[35]	72.5%	68.5%	69.7%	68.1%	79.0%	77.0%	77.3%	75.5%
**Wang 2018**[36]	80.6%	71.0%	76.0%	67.0%	85.7%	87.1%	85.7%	81.0%
**Huang 2021**[30]	81.0%	32.5%	NR	NR	86.2%	64.3%	NR	NR
**Sanders 2021**[37]	63.0%	69.0%	51.0%	64.0%	66.0%	79.0%	54.0%	73.0%
**Median**	80.6%	71.0%	76.0%	68.1%	81.0%	79.0%	79.8%	74.7%
**Weighted mean**	74.0%	70.3%	72.0%	67.3%	81.1%	83.1%	79.1%	77.0%

NR: Not reported. PAO-RT: para-aortic radiotherapy.

## Discussion

Locally advanced cervical cancer with pelvic lymph node involvement is sometimes associated with occult para-aortic lymph node metastases (PAO-LNM). However, in the current era in which staging techniques have improved and the addition of chemotherapy to radiotherapy is the standard of care, the role of PAO-RT for patients with pelvic lymph node involvement has not been clearly established. In this meta-analysis, pooling of aHRs reported in 3 studies provided evidence that extending the radiation field to cover para-aortic lymph nodes may reduce the risk of disease recurrence over time by 13%, with no evidence of heterogeneity across studies. These pooled HR estimates were adjusted for other prognostic factors within the individual studies. Given the size of the observed beneficial effect of PAO-RT on DFS (pooled aHR 0.87), PAO-RT may be regarded as a tool to improve nodal control, and potentially also distant metastatic control and overall survival as a consequence. The latter suggestion, however, requires additional research ideally in a prospective randomized setting.

The finding that PAO-RT improves DFS raises the question whether staging procedures could be improved for better selection of patients who will benefit. Most studies included in this review used CT or MRI for lymph node staging. However, these imaging modalities used to detect para-aortic lymph node metastases have limited sensitivity [38], [39], [40]. In addition, PET-CT was shown to have a low negative predictive value for detecting PAO-LNM [12]. In line with this observation, some studies have reported high rates of upstaging after surgical staging of para-aortic lymph nodes compared to imaging only [12], [41], [42]. In addition, a large 2010 multi-centre cohort analysis by the FRANCOGYN study group showed that nodal surgical staging had a positive impact on survival in locally advanced cervical cancer patients with no evidence of PAO-LNM on imaging who were treated with chemoradiotherapy [43]. However, surgical staging remains controversial because it has particularly not appeared able to improve the poor survival outcomes of patients with PAO-LNM greater than 5 mm, it could lead to surgical morbidity and a delay in initiating treatment [44], [45].

In the majority of cases, PAO-LNM is correlated with positive pelvic lymph nodes as skip metastases are rare [7], [46]. Multiple studies demonstrated that cervical cancer patients with pelvic lymph node involvement should be considered at high risk of developing para-aortic lymph node relapse and thus might benefit from elective PAO-RT [44], [47], [48]. In accordance, the currently ongoing EMBRACE-II multicenter study prescribes addition of PAO-RT in patients with high-risk disease, defined as ≥ 1 pathologic node at the common iliac level or above, or ≥ 3 pathologic nodes at any level [11]. Other possible risk factors for developing PAO-LNM might include age, tumour size, FIGO stage and squamous cell carcinoma antigen (SCCA) elevation [49].

Regarding the toxicity of PAO-RT, some recent studies have shown acceptable toxicity rates (6–10% acute grade ≥ 3 gastro-intestinal (GI) toxicity and 6.5% late grade ≥ 3 GI toxicity) after PAO-RT with intensity-modulated radiotherapy (IMRT) [50], [51], [52]. Conversely, older RTOG trials reported higher rates of toxicity (14–49% grade ≥ 3 toxicity) [6], [53]. However, this may be due to the used 2D radiotherapy technique that is now considered obsolete [54]. Modern radiotherapy techniques, including IMRT and image-guided adaptive radiotherapy, are associated with lower rates of acute and late toxicity [51]. The overall volume irradiated to 43 Gy is strongly associated with bowel morbidity, and can be lowered most effectively by omitting PAO-RT, but in case of PAO-RT by using IMRT instead of 3D conformal RT [11], [20]. As such, in light of the findings of this meta-analysis, individualized PAO-RT using modern radiotherapy techniques can be considered in contemporary practice with acceptable toxicity risks.

Future directions in the management of PAO-LNM from cervical cancer include optimization of both systemic therapy and radiotherapy and patient selection for PAO-RT. A large recent trial demonstrated that systemic therapy intensification by adjuvant chemotherapy after standard cisplatin-based chemoradiotherapy in patients with locally advanced cervical cancer unfortunately did not improve DFS or OS [55]. Intensification through the addition of immunotherapy as an induction, concurrent and/or adjuvant regimen with chemoradiotherapy is under investigation in multiple ongoing studies (e.g. ATEZOLACC [NCT03612791], COLIBRI [NCT04256213], NiCOL [NCT03298893]), including 2 large multicenter phase III randomized trials (i.e. CALLA [NCT03830866] and GOG-3047 [NCT04221945]). RT may be further optimized by reducing the dose to organs-at-risk using intensity-modulated proton therapy (IMPT), which might yield particular benefits in patients undergoing PAO-RT. This approach of proton therapy is currently under investigation, for example in the PROTECT trial [56]. Patient selection for PAO-RT may be improved by recently developed nomograms that predict PAO nodal failure [57], [58]. In addition, ongoing research is exploring if the prediction accuracy of such nomograms could be improved by studying molecular tumor profiles or extracting imaging features with radiomics or deep learning techniques [59], [60], [61].

A few limitations apply to this review. The findings are impacted by the biases and limitations of the included studies. First, unfortunately not all studies comparing PAO-RT to no PAO-RT provided adjusted HRs, which is required for formal meta-analysis. Second, the 3 studies included in the meta-analysis adjusted for prognostic factors differently, which may have impacted the adjusted HRs of the effect of PAO-RT on DFS to some extent. Third, due to the small number of studies appropriate for quantitative meta-analysis, and the lack of individual patient data availability, no subgroup analyses could be performed. Fourth, as for the literature analysis, some articles lacked detailed information on pelvic and/or para-aortic lymph node involvement, which may have led to unreliable results since an unreported variation in the proportions of positive nodes could cause different outcomes.

In conclusion, this meta-analysis of 3 studies suggests a significant association between elective para-aortic lymph node irradiation and improved DFS in cervical cancer patients with pelvic lymph node metastases who undergo primary (chemo)radiotherapy (pooled aHR 0.86). Literature review of 8 additional studies that compared PAO-RT to no PAO-RT (but were not eligible for quantitative synthesis) confirmed this trend of a beneficial impact on DFS by PAO-RT. One approach to deal with this finding includes the recommendation of the EMBRACE-II study group to add PAO-RT in patients at high risk of PAO-LNM [11]. However, the role of elective PAO-RT in the era of concurrent chemoradiotherapy remains debatable in the absence of a large-scale randomized trial with modern radiotherapy techniques.

## Declaration of Competing Interest

The authors declare that they have no known competing financial interests or personal relationships that could have appeared to influence the work reported in this paper.
